# The study of dry biological valve crosslinked with a combination of carbodiimide and polyphenol

**DOI:** 10.1093/rb/rbaa049

**Published:** 2020-12-03

**Authors:** Li Yang, Shuang Xie, Kailei Ding, Yang Lei, Yunbing Wang

**Affiliations:** National Engineering Research Center for Biomaterials, Sichuan University, No. 29 Wangjiang Road, Chengdu 610064, China

**Keywords:** dry biological valve, carbodiimide, polyphenol, non-glutaraldehyde

## Abstract

The glutaraldehyde crosslinked pericardium has been used in bioprosthetic valves for about 50 years. However, problems such as glutaraldehyde residue and calcification still exist in current commercial products. Non-glutaraldehyde crosslinked dry valve is an important strategy to solve those problems. In this study, a non-glutaraldehyde crosslinked dry biological valve material was obtained by the combined crosslinking of carbodiimide (EDC) and polyphenol. The results showed that the comprehensive properties of EDC and curcumin crosslinked pericardium were superior to glutaraldehyde crosslinked pericardium, including unfolding property, anti-calcification, cytotoxicity, anticoagulant properties, mechanical properties, enzyme degradation resistance and thermal shrinkage temperature. EDC and curcumin crosslinked dry pericardium could flatten after being folded at 40°C for 3 days while glutaraldehyde crosslinked pericardium could not. The calcification of pericardium treated with EDC and curcumin was 1.21 ± 0.36 mg/g in rats after 60 days’ subdermal implantation, much lower than that of glutaraldehyde treated control group (22.06 ± 3.17 mg/g).

## Introduction

Artificial heart valves can be used to treat patients with heart valve disease. It is predicted that the market demand for interventional heart valve products will exceed that of interventional coronary stents in the next five years. Currently, commercial bioprosthetic heart valves are mostly made of glutaraldehyde crosslinked pericardia. Bioprosthetic valves have been used in a large number of successful clinical applications in recent decades. However, they still have a short lifespan of 10–15 years. In addition, current bioprosthetic valves need to be stored in glutaraldehyde solution, and this wetting state of preservation further raises the issue of residual glutaraldehyde. At the same time, the wet valve needs to be instantly cleaned and installed before the valve replacement operation. These processes are relatively complicated, which cannot meet the emergency needs for the valve replacement operation.

Dry valve is a new technique for preparing and preserving valves. Valve materials are directly dried and preserved after crosslinking treatments. It is no longer necessary to keep the valve in glutaraldehyde solution. Thus, steps such as preoperative cleaning and installation can be avoided, which can reduce the residual glutaraldehyde and shorten the preoperative preparation time of the instrument [1]. In dry valve materials’ research, our initial attempt to prepare glutaraldehyde crosslinked dry pericardium using different glutaraldehyde concentrations, temperatures, and time, failed in folding test. Glutaraldehyde crosslinked dry valve could not flatten in water.

In order to solve those problems, it is of great significance to develop non-glutaraldehyde crosslinked dry valve materials. Since glutaraldehyde is not used, all problems caused by the aldehyde group residue of glutaraldehyde can be avoided from the source. The valve does not need to be stored in wet state by solution, but can be sterilized by the method of ethylene oxide to retain in dry state instead. Thus, doctors can open sterilized bags and use them immediately.

The use of carbodiimide (EDC) and polyphenols in the crosslinking of bioprosthetic valves has been studied [2–6]. EDC is a kind of compound containing C–N double bond, which has higher reactivity and can activate carboxyl groups on the pericardium. The amino and carboxyl groups on the pericardium can be utilized to achieve the crosslinking based on amide formation process. N-hydroxysuccinimide (NHS) could further enhance the crosslinking efficiency of EDC [2, 5]. EDC crosslinking can effectively enhance the stability of collagen, but the crosslinking effect on elastin is limited [2]. Polyphenolic compounds, including curcumin and procyanidins, can achieve pericardium crosslinking through hydrogen bonding [3–6]. It has been reported that polyphenols can effectively stabilize elastin. Curcumin is a natural phenolic antioxidant. Its main chain is unsaturated aliphatic group and aromatic group. Procyanidins are polyphenols synthesized by condensation of flavane-3-alcohol monomer, which also has antioxidant activity. It has been reported that the pericardium crosslinked with curcumin or procyanidin exhibited satisfactory anti-calcification property [3, 5].

Therefore, the aim of this paper is to investigate the anti-enzyme degradation stability, thermal shrinkage temperature, mechanical properties, anti-compression damage ability, anti-coagulation ability, cytotoxicity, calcification and other properties of bioprosthetic valves through the combined crosslinking of carbodiimide and polyphenol. A preparation method of non-glutaraldehyde crosslinked dry valve material was obtained.

## Materials and methods

### Materials

Fresh porcine pericardium was kindly provided by Venus Medtech Inc (Hangzhou, China). Disodium ethylenediamine tetraacetic acid (EDTA), sodium dodecylsulphate (SDS), glutaraldehyde (GLUT), 1-ethyl-3-(3-dimethylaminopropyl) carbodiimide (EDC), N-hydroxysuccinimide (NHS) and procyanidine (PC) were purchased from Huaxia Reagent (Chengdu, China). Collagenase II was purchased from Sigma-Aldrich (St. Louis, MO, USA). Elastase and curcumin (CC) were purchased from Macklin Biochemical Co., Ltd (Shanghai, China). Cell counting cit-8 (CCK8) assay kit was bought from Beyotime Biotechnology Co., Ltd. (Shanghai, China).

### Pericardia treatments

Pericardia were decellularized by 0.1% EDTA plus 0.1% SDS for 24 h [7]. Then pericardia were crosslinked with five different methods: (i) GLUT; (ii) CC; (iii) EDC/CC; (iv) PC and (v) EDC/PC. Crosslinked pericardia were soaked in the solution of 80% ethanol plus 20% glycerol for 4 h and then air-dried for preservation.

- GLUT: Decellularized tissues were soaked in 0.625% GLUT for 24 h at room temperature [8].
- CC: Tissues were soaked in the solution of 0.1 mM curcumin with 1:9 (Ethanol: phosphate buffer saline (PBS)) ethanol and PBS mixed solutions at room temperature for 24 h [6].
- EDC/CC: Pericardia were then treated with 60 mM EDC and 12 mM NHS mixture for 24 h [2, 9]. Then, the material was soaked in the solution of 0.1 mM curcumin with 1:9 (Ethanol: PBS) ethanol and PBS mixed solutions at room temperature for 24 h.
- PC: The material was soaked in the PBS solution of 10 mg/mL procyanidine at room temperature for 24 h [3].
- EDC/PC: Pericardia were then treated with 60 mM EDC and 12 mM NHS mixture for 24 h [2, 9]. Then, the material was soaked the PBS solution of 10 mg/mL procyanidine at room temperature for 24 h.

### Pre-crimping test

Samples (3 × 3 cm) were folded and put into a 5 mm diameter catheter for 3 days at 40°C. The size of the catheter for the existing interventional aortic valve is approximately 5 mm. The dry valves need to be pre-loaded into the catheter and then sterilized with ethylene oxide for approximately 3 days at approximately 40°C. The samples were then put in saline to take photos and videos.

### *In vivo* rat subdermal implantation model

All animal experiments were approved by the Medical Ethics Committee of Sichuan University [10]. 1 × 1 cm samples were put in 50 g male SD rats for 60 days (*N* = 6, six samples in each group, two samples per rat). Then, calcium contents were tested by inductively coupled plasma-atomic emission spectrometry (ICP-AES).

### Cytotoxicity test

The preparation of the material extracts was according to ISO 10993-5:2009, Biological evaluation of medical devices—Part 5: Tests for *in vitro* cytotoxicity [11]. Samples were soaked in cell culture medium at 37°C for 48 h in a tissue-weight to volume ratio of 0.2 g/mL. Then, L929 fibroblasts were cultured with the extracts for 1 day to test the cell viability.

### Platelet and whole blood adhesion tests

The thrombogenic properties of the tissues were assessed using platelet and whole blood adhesion tests as described before [12, 13]. Samples were put in 96-well plate with the addition of platelet or whole blood for 1 h. Then the samples were washed and dehydrated for scanning electron microscope test.

### Suture pull-out test and uniaxial tensile test

Samples with 22 × 3 mm rectangular size were prepared (*N* = 4). For the suture pull-out test [2], a single suture was passed through the sample and then attached to the clamp. The thickness of the sample was measured. The maximum force and extensibility were analysed by a tensile tester (BioTester, 10 N load cell; CellScale, Waterloo, Ontario, Canada). The mechanical properties of the pericardium were non-isotropic, so the material used for uniaxial mechanical testing was in the parallel direction of collagen fibers. The direction of the sample was confirmed under the light and magnifying glass. The supplement information had the methods and results of biaxial mechanical test, which further confirmed the non-isotropy of the material.

### Enzymatic degradation *in vitro*

Samples (about 10 mg each, *N* = 4) were freeze-dried and weighted. Then the samples were incubated in collagenase (75 U/ml) or porcine pancreatic elastase (30 U/ml) for 1 day at 37°C. [2]. The percent weight loss was calculated.

### Differential scanning calorimetry (DSC)

Differential scanning calorimetry was used to measure the thermal shrinkage temperature of tissues [2]. Dry samples (5–10 mg for each, *N* = 4) were tested by a DSC 2920 under N_2_ atmosphere (TA Instruments, Newcastle, DE). Samples were equilibrated at 40°C, and heated at the rate of 10°C/min up to 100°C. The thermal shrinkage temperature was recorded as the maximum value of the endotherm peak.

### Statistical analysis

Quantitative data are shown as mean ± standard deviation (SD). Single-factor analysis of variance (ANOVA) was used for statistical test.

## Results

### Pre-crimping test

**Figure 1.**
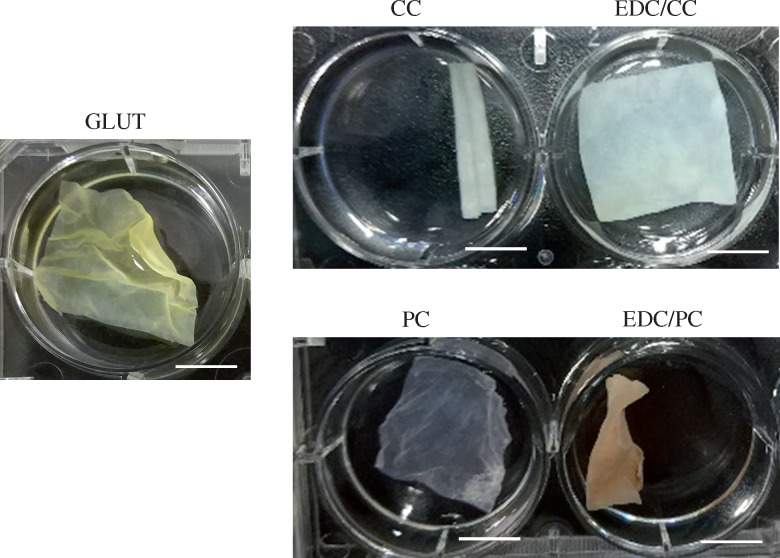
Representative photos for the tissues (3 cm × 3 cm) pre-crimped in catheter after put into PBS are shown. Scale bar: 1 cm. The dry valve in the glutaraldehyde (GLUT) treatment group had obvious creases while the EDC/CC treatment group flattened without creases or damage.

The pre-crimping test results showed that the dry valve in the glutaraldehyde treatment group could not completely flatten in water after being folded at 40°C for 3 days, and the creasing was relatively obvious (Fig. 1, Supplementary Video S1). Obvious creases were observed on the glutaraldehyde treated group, indicating that crimping procedure caused damage to the material. CC treatment group was also unable to flatten, showing tubular curly state. The EDC/CC group flattened without creases or damage within 1 min (Supplementary Videos S2 and S3). PC or EDC/PC group had obvious creases (Supplementary Videos S4 and S5).

### *In vivo* rat subdermal implantation model

**Figure 2.**
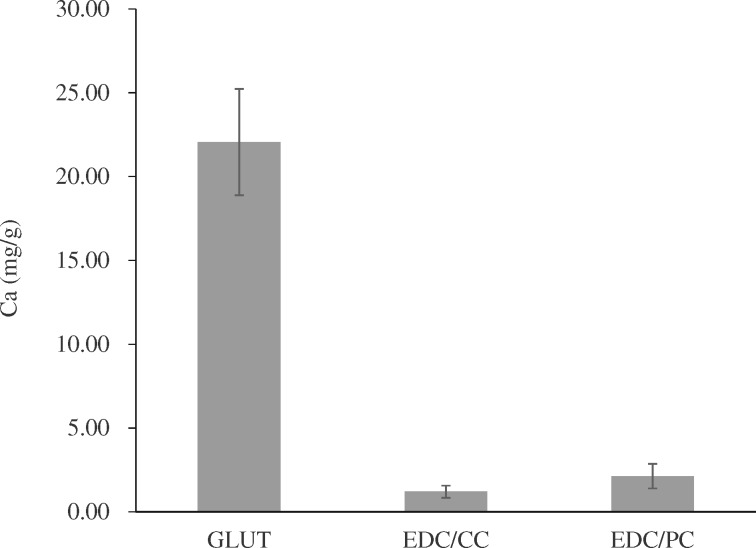
Calcium contents of each explant were calculated. *N* = 6. The calcification content of EDC/CC and EDC/PC group was much lower than that of GLUT group.

The rat subdermal implantation results showed that calcification contents were 22.06 ± 3.17 mg/g in glutaraldehyde group, 1.21 ± 0.36 mg/g in EDC/CC group and 2.14 ± 0.74 mg/g in EDC/CC group (Fig. 2). The calcification content of EDC/CC and EDC/PC group was much lower than that of glutaraldehyde group.

### Cytotoxicity test

**Figure 3.**
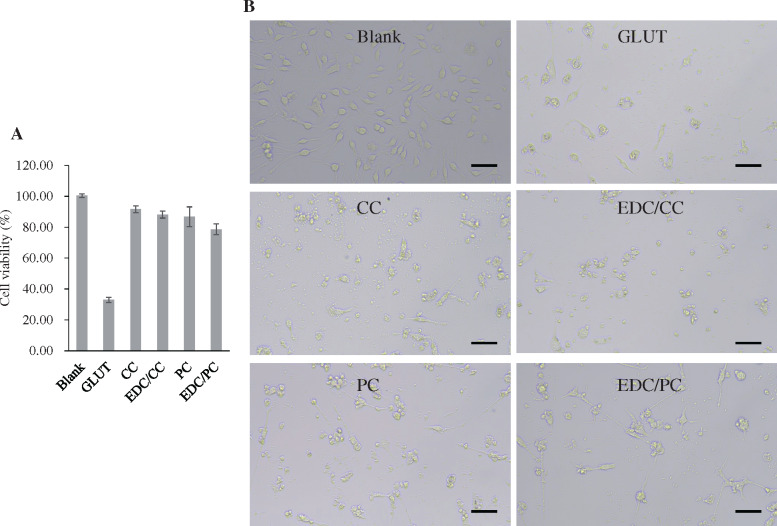
A. Cell viability in each group. *N* = 3 (three independent experiments). **B.** Microscopic images (200×) of L929 cells are shown. Scale bar = 100. The cytotoxicity of polyphenols or polyphenols combined with EDC was significantly lower than that of glutaraldehyde.

The cytotoxicity test results showed that the cell viability of glutaraldehyde group was 32.94 ± 1.73%, that of CC and EDC/CC treatment group was 91.67 ± 2.25% and 88.19 ± 2.25%, respectively, and that of PC and EDC/PC treatment group was 86.76 ± 6.41% and 78.68 ± 3.57%, respectively (Fig. 3). The cell viability of CC, EDC/CC, PC, EDC/PCC treatment group was significantly higher than that of glutaraldehyde treatment group. This indicated that the cytotoxicity of polyphenols or polyphenols combined with EDC was significantly lower than that of glutaraldehyde. Images from white light microscopes tend to look similar.

### Platelet and whole blood adhesion tests

**Figure 4.**
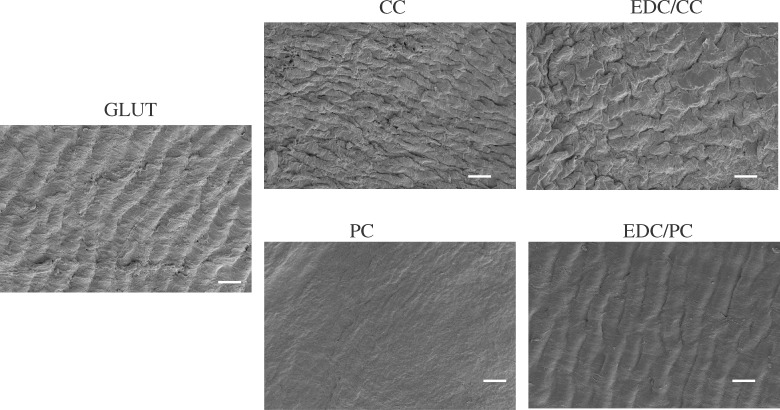
Representative scanning electron microscope images of adherent platelets on the samples. Scale bar: 20 µm (2000×). There was more adhered platelet in the glutaraldehyde treatment group, and almost no adhered platelet was seen in the CC, EDC/CC, PC, or EDC/PCC treatment group.

**Figure 5.**
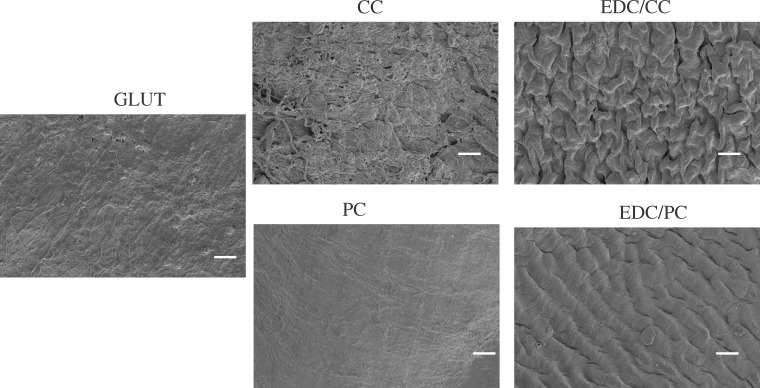
Representative scanning electron microscope images of adherent whole blood on the samples. Scale bar: 20 µm (2000×). The results of whole blood adhesion tests were consistent with the trend of platelet adhesion.

The results of the platelet adhesion test showed that there was more adhered platelet in the glutaraldehyde treatment group, and almost no adhered platelet was seen in the CC, EDC/CC, PC or EDC/PCC treatment group (Fig. 4). This indicated that the blood compatibility of polyphenols or polyphenols combined with EDC was better than that of glutaraldehyde group. The results of whole blood adhesion tests were consistent with the trend of platelet adhesion (Fig. 5).

### Suture pull-out test

**Figure 6.**
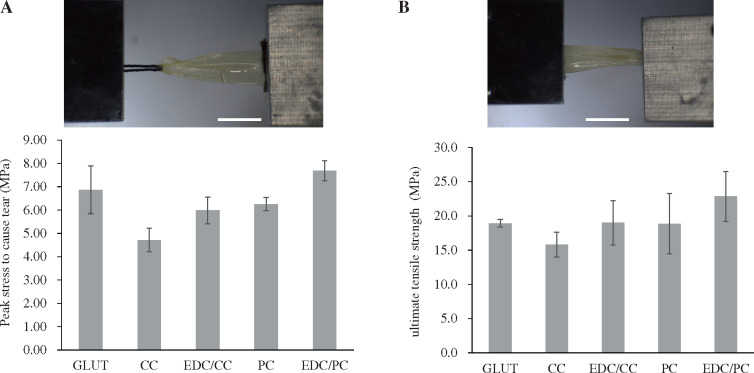
Peak stress required to cause tearing (**A**) and ultimate tensile strength of pericardium samples (**B**) is shown. *N* = 4 (four independent experiments). Scale bar: 1 cm. The peak stress to cause tear of CC group was lower than that of glutaraldehyde group, while EDC/CC, PC, or EDC/PC treatment group was close to glutaraldehyde treatment group. The maximum tensile strength in CC group was lower than that in glutaraldehyde group, while EDC/CC, PC, EDC/PC treatment groups were close to glutaraldehyde treatment group.

The suture pull-out test results showed that the peak stress to cause tear were 6.87 ± 1.02 MPa in the glutaraldehyde group, 4.71 ± 0.51 MPa and 5.99 ± 0.58 MPa in CC and EDC/CC groups, respectively, and 6.25 ± 0.29 MPa and 7.69 ± 0.43 MPa in PC and EDC/PC groups, respectively (Fig. 6A). The peak stress to cause tear of CC group was lower than that of glutaraldehyde group. EDC/CC, PC or EDC/PC treatment group was close to glutaraldehyde group.

### Uniaxial tensile test

The uniaxial tensile test results showed that the maximum tensile strengths were 18.9 ± 0.5 MPa in the glutaraldehyde group, 15.8 ± 1.8 MPa and 19 ± 3.2 MPa in CC and EDC/CC groups, respectively, 18.9 ± 4.4 MPa and 22.9 ± 3.6 MPa in PC and EDC/PC groups, respectively (Fig. 6B). The maximum tensile strength in CC group was lower than that in glutaraldehyde group. EDC/CC, PC EDC/PC treatment groups were close to glutaraldehyde treatment group.

**Figure 7.**
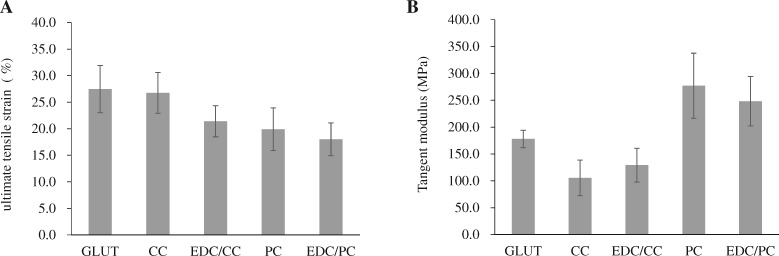
Ultimate tensile strain of pericardium samples (**A**) and tangent modulus (**B**) are shown. *N* = 4 (four independent experiments). The maximum tensile strain of CC group was similar to that of glutaraldehyde group, while EDC/CC, PC, or EDC/PC treatment group was slightly lower than glutaraldehyde treatment group.

The maximum tensile strain test results showed that the maximum tensile were 27.5 ± 7.7% in glutaraldehyde group, 26.8 ± 6.7% and 21.4 ± 5.1% in CC and EDC/CC groups, respectively, 19.9 ± 6.9% and 18 ± 5.4% in PC and EDC/PC groups, respectively (Fig. 7A). The maximum tensile strain of CC group was similar to that of glutaraldehyde group. EDC/CC, PC or EDC/PC treatment group was slightly lower than glutaraldehyde treatment group.

The tangent modulus test results showed that the tangent moduli were 177.9 ± 27.8 MPa in glutaraldehyde group, 105.5 ± 57.2 MPa and 129.1 ± 54.5 MPa in CC and EDC/CC treatment groups, respectively, and 277.1 ± 104.9 MPa and 248.3 ± 79.7 MPa in PC and EDC/PC treatment groups, respectively (Fig. 7B). The tangent modulus of CC or EDC/CC treatment group was close to that of glutaraldehyde treatment group. However, the tangent modulus of PC or EDC/PC treatment group was higher than that of glutaraldehyde treatment group.

### Enzymatic degradation in vitro

**Figure 8.**
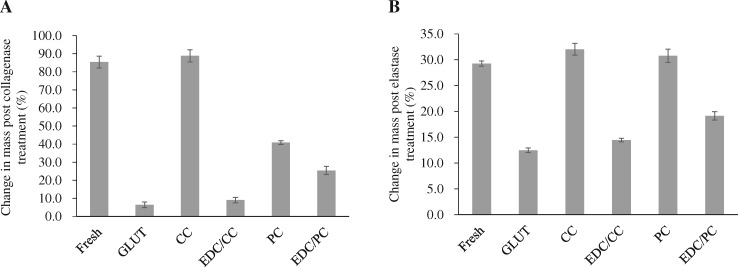
Collagenase and elastase enzymatic degradation *in vitro*. Change in mass post collagenase (**A**) and elastase (**B**) treatment. *N* = 4 (four independent experiments). The protective effect of the EDC/CC or EDC/PC combination treatment group on collagen was similar to that of the glutaraldehyde crosslinking group.

Experimental results of collagenase degradation showed that the weight loss rate of fresh pericardium was 85.38 ± 3.26%, and glutaraldehyde was 6.44 ± 1.59%. CC and EDC/CC groups were 88.84 ± 3.39% and 9.06 ± 1.46%, respectively, while PC and EDC/PC groups were 40.85 ± 1.02% and 25.43 ± 2.27%, respectively (Fig. 8A). The weight loss rate of collagenase degradation in CC or PC treated group was higher than that in glutaraldehyde treated group. The protective effect of the EDC/CC or EDC/PC combination treatment group on collagen was similar to that of the glutaraldehyde crosslinking group.

Experimental results of elastase degradation showed that the weight loss rates of fresh pericardium and glutaraldehyde were 29.26 ± 0.51% and 12.48 ± 0.44%, respectively; 32.02 ± 1.13% and 14.44 ± 0.36% in CC and EDC/CC groups, respectively, 30.78 ± 1.29% and 19.14 ± 0.83% in PC and EDC/PC groups, respectively (Fig. 8B). The weight loss rate of elastase degradation in CC or PC treated group was higher than that in glutaraldehyde treated group. The protective effect of EDC/CC or EDC/PC treatment group on elastin was similar to that of glutaraldehyde crosslinking group.

### Differential scanning calorimetry

**Figure 9.**
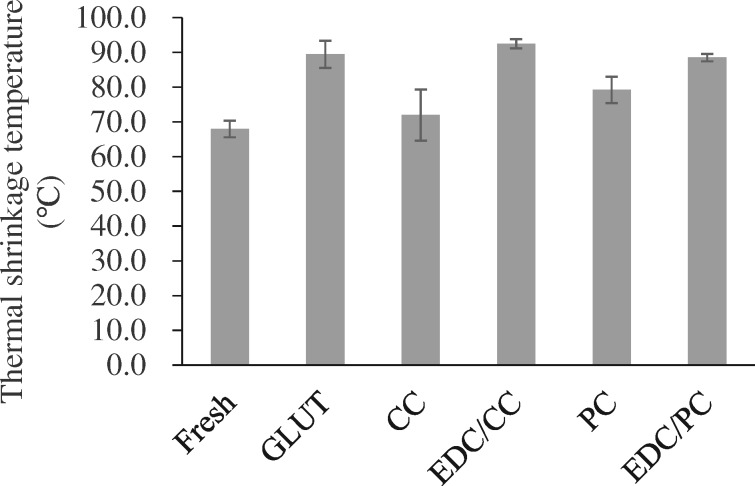
Thermal shrinkage temperature of each group. *N* = 4 (four independent experiments). The thermal shrinkage temperature of the EDC/CC or EDC/PC treatment group was close to that of the glutaraldehyde crosslinking group.

DSC results showed that the thermal shrinkage temperature of fresh pericardium was 68 ± 2.4°C, and glutaraldehyde crosslinked pericardium was 89.5 ± 3.9°C. CC and EDC/CC treatment groups were 71.9 ± 7.4°C and 92.5 ± 1.4°C, respectively, while PC and EDC/PC treatment groups were 79.2 ± 3.8°C and 88.5 ± 1.1°C, respectively (Fig. 9). The thermal shrinkage temperature of CC or PC treated group was lower than that of glutaraldehyde treated group. The thermal shrinkage temperature of the EDC/CC or EDC/PC treatment group was close to that of the glutaraldehyde crosslinking group.

## Discussion

At present, a variety of non-glutaraldehyde crosslinking methods can be found in the literature, including chemical crosslinkers such as carbodiimide [2, 5], epoxides [14, 15], free radical polymerization crosslinkers [4, 16], and natural crosslinkers such as curcumin [6], procyanidins [3, 5], genipin [17, 18], tannins [19], etc. However, currently, all the biological valve products in clinical use are prepared by crosslinking with glutaraldehyde, and almost no pericardial materials with non-glutaraldehyde crosslinking have been used for commercial biological valve products. This indicates that the non-glutaraldehyde crosslinking method still faces great challenges. The crosslinking of glutaraldehyde for biological valves has been used in clinical practice since the 1970s, and its safety and efficacy have been fully verified in clinical application in the past 50 years. In addition, preassembly of biological valves’ damage still exists. How to improve the ability of the dry biological valve to withstand folding and compression, as well as the ability to restore its original shape, is still a problem to be solved.

In our study, we used two combined crosslinking methods of carbodiimide and polyphenol compounds. Among them, the carbodiimide and curcumin combination treatment group showed better comprehensive properties (Table 1).

**Table 1.** Main properties comparison of non-glutaraldehyde pericardium and glutaraldehyde pericardium

	EDC/CC non-glutaraldehyde pericardium vs glutaraldehyde pericardium
Dry valve unfolding	Much better
Anti-calcification	Much better
Cytotoxicity	Better
Anti-coagulation	Better
Peak stress to cause tear	Similar
Maximum tensile strength	Similar
Maximum tensile strain	Similar
Resistance to enzyme degradation	Similar
Thermal shrinkage temperature	Similar

The crimping process would damage the valve material [20, 21]. Pre-crimped dry biological valves need to be refolded and sterilized with ethylene oxide at approximately 40°C for 24–72 h under pressure, which requires good pressure tolerance and good toughness. In the pre-crimping test, the pericardial tissue was folded and squished together before being plugged into a catheter, so the material was squeezed and deformed in all directions to simulate the loading of the transcatheter biological valve. From the experimental results, the glutaraldehyde control group showed certain creases and damage in all directions after folding and pressing, without obvious anisotropy. The EDC/CC treatment group was able to recover its original shape under folding pressure, rapidly flattening in water with almost no creases, suggesting that the non-glutaraldehyde system crosslinked pericardium is more suitable for dry valve (Fig. 1). The formation of stable crosslinks by glutaraldehyde is formed by either the combination of two intermediate Schiff bases or the addition of another ε-amino group to the double bond [22]. Carbodiimide can crosslink neighboring carboxyl and amine groups and form amide bonds [23]. Carbodiimide crosslinked pericardia were softer. Our results confirmed that carbodiimide and curcumin group had lower modulus compared to the glutaraldehyde group (Fig. 7B). This might be the reason why EDC/CC crosslinked dry pericardia had better unfolding capability.

Calcification of biological valves is an old problem that has been studied for a long time. Calcification mechanism includes multiple factors, including glutaraldehyde residue, elastin component degradation, mechanical damage and so on [24, 25]. Calcification was significantly reduced in the EDC/CC treatment group compared to the glutaraldehyde treatment group (Fig. 2). The reason for this may be that the EDC/CC treatment group does not contain aldehyde groups, which can protect the pericardial components such as elastin.

According to international standards on medical devices and materials, biocompatibility including cytotoxicity should be investigated for biological valve as a long-term implanted device. The EDC/CC treatment group had less cytotoxicity than the glutaraldehyde treatment group, indicating better biocompatibility (Fig. 3).

The working environment of biological valve needs to be exposed to blood for a long time, so we need to investigate its anticoagulant ability. Although biological valves have better anticoagulant properties than mechanical valves and do not require long-term use of anticoagulant drugs, chronic mild coagulation has been reported. GLUT fixed pericardia showed acceptable hemocompatibility [26]. The EDC/CC treatment group had fewer platelets and less whole-blood adherence than the glutaraldehyde treatment group, suggesting that it may have better anticoagulant properties (Figs 4 and 5).

The maximum tensile strength is related to the quality, thickness, decellularization process and the crosslinking degree of the pericardial materials. The maximum tensile strain of the pericardial material is expected to be as small as possible. Studies have shown that the smaller the maximum tensile strain, the lower the probability of irreversible deformation of the valve in the long-term bending state. The EDC/CC treatment group has the maximum tensile strength and the maximum tensile strain equivalent to the glutaraldehyde crosslinked materials, which can meet the requirements of existing products (Fig. 6).

The anti-enzyme degradation properties can predict the tolerance of biological valves to various enzymes in the blood contact environment after implantation, so as to predict the durability to some extent. Compared with glutaraldehyde treatment group, the EDC/CC treatment group had similar anti-collagenase degradation and anti-elastase degradation properties. The protective effect of curcumin or procyanidin alone on collagen or elastin was limited (Fig. 8). It has been reported that curcumin or proanthocyanidin alone crosslinking has a good protective effect on collagen [3, 6]. Our results are inconsistent with those reported in the existing literature. Polyphenols can specifically bind to hydrophobic regions in proline-rich proteins [19, 27].

Thermal shrinkage temperature can further indirectly reflect the stability of collagen in biological valves. In terms of thermal shrinkage temperature, the EDC/CC treatment group was comparable to the glutaraldehyde treatment group (Fig. 9). In previous studies, biological valves treated with EDC alone had lower thermal contraction temperatures than those treated with glutaraldehyde [2]. Our results show that it is more effective to combine the two methods.

## Conclusions

This study adopts the EDC and polyphenol combined method for the preparation of dry biological valve materials. Compared with the glutaraldehyde crosslinked pericardium, our non-glutaraldehyde crosslinked pericardium has better comprehensive properties, including better dry valve unfolding capability, anti-calcification property, cell toxicity, and blood compatibility, as well as similar mechanical properties, enzyme degradation resistance and thermal shrinkage temperature. This new biological valve material has great potential for clinical application.

## Supplementary data

Supplementary data are available at *REGBIO* online.

## Supplementary Material

rbaa049_Supplementary_Data
