# Supplementary material for: The study of dry biological valve crosslinked with a combination of carbodiimide and polyphenol
Source: Regen Biomater. 2020 Dec 3;8(1):rbaa049. doi: 10.1093/rb/rbaa049 (PMC7947589; doi:10.1093/rb/rbaa049)
Supplement: rbaa049_Supplementary_Data [file rbaa049_supplementary_data.zip › suppl_data/SI 200908-1449.docx]

**Supplement Information**

**The study of dry biological valve crosslinked with a combination of carbodiimide and polyphenol**

**Li Yang, Shuang Xie, Kailei Ding, Yang Lei*, and Yunbing Wang**

National Engineering Research Center for Biomaterials, Sichuan University, No. 29 Wangjiang Road, Chengdu 610064, China

**^*^**Correspondence authors at: National Engineering Research Center for Biomaterials, Sichuan University, No. 29 Wangjiang Road, Chengdu 610064, China. Tel: +86 28 85415280; Fax: +86 28 85410246; E-mail: E-mail: [leiyang@scu.edu.cn](mailto:leiyang@scu.edu.cn) (Dr. Yang Lei)

**Materials and Methods**

*Biaxial tensile test*

Samples from EDC/CC treatment group was cut into 1 cm × 1 cm squares (N = 4). The fiber direction was made parallel or perpendicular to the edge. The sample was then placed on a rubber pad and secured to the tensile tester (BioTester, 10 N load cell; CellScale, Waterloo, Ontario, Canada) in PBS solution [1]. The tissue was passed through by the hooks of each side of the sample, and the four edges were evenly distributed. The horizontal was defined as the X direction and the vertical was defined as the Y direction. The force loading test protocol was used to maximize the load force (approximately 1500 N ~ 2500 N, depending on the material) while ensuring that the sample was not damaged. The force value loaded in X direction and Y direction remained 1:1. After the strain was derived by image tracking, the stress-strain curve of the sample was calculated.

**Results**


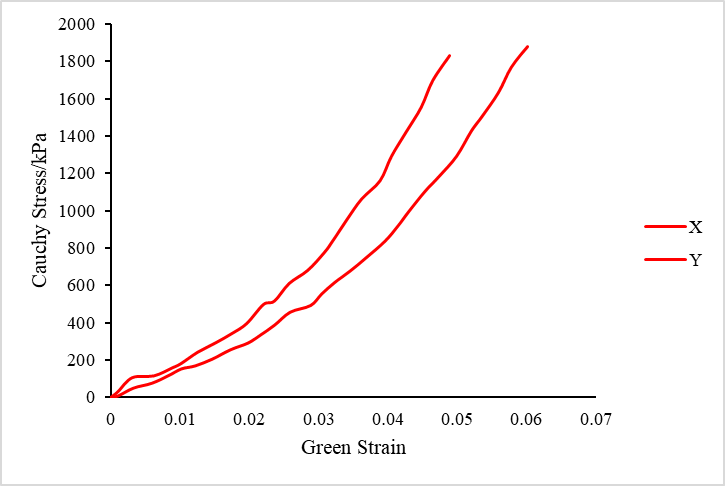


Figure S1. Biaxial tensile stress-strain curve.

The biaxial stretching results show that the pericardium has different elastic modulus in X and Y perpendicular directions, indicating its non-isotropy (Figure S1).

**References**

1. Caballero, A., et al., *Evaluation of transcatheter heart valve biomaterials: Biomechanical characterization of bovine and porcine pericardium.* J Mech Behav Biomed Mater, 2017. **75**: p. 486-494.
