# Supplementary material for: The study of dry biological valve crosslinked with a combination of carbodiimide and polyphenol
Source: Regen Biomater. 2020 Dec 3;8(1):rbaa049. doi: 10.1093/rb/rbaa049 (PMC7947589; doi:10.1093/rb/rbaa049)
Supplement: rbaa049_Supplementary_Data [file rbaa049_supplementary_data.zip › suppl_data/cover letter-200719-1013.docx]

**Cover letter**

Date: July 19, 2020

Dear editors:

We would like to submit our manuscript entitled “The study of dry biological valve crosslinked with a combination of carbodiimide and polyphenol".

The crosslinked pericardium of glutaraldehyde has been used in biological valves for about 50 years. However, current products have problems such as glutaraldehyde residue, calcification, limited durability, and need to be soaked in harmful solutions such as glutaraldehyde solution for preservation. Non-glutaraldehyde crosslinked dry biological valve is an important strategy to solve the above problems. The dry valve material can be sterilized with the delivery system as a whole. Doctors can open the package and use it immediately, satisfying the need for emergency valve replacement surgery and reducing the surgical risk for patients.

In this study, a non-glutaraldehyde crosslinked dry biological valve material with good comprehensive properties was obtained by the combined crosslinking of carbodiimide (EDC) and polyphenol. The results showed that EDC and curcumin crosslinked pericardia achieved or exceeded glutaraldehyde crosslinked pericardia in dry valve unfolding property, anti-calcification, cytotoxicity, anticoagulant properties, mechanical properties, enzyme degradation resistance, and thermal shrinkage temperature. This is a new kind of biological valve material with potential of clinical applications.

All authors were fully involved in the study and preparation of the manuscript and claim none of the material in the paper has been published or is under consideration for publication elsewhere. There is no conflict of interests associated with this publication.

Thanks for your consideration.

Suggested reviewers：

William Wagner, PhD, University of Pittsburgh, [wagnerwr@upmc.edu](mailto:wagnerwr@upmc.edu)

William Wagner is expert in cardiovascular biomaterials

Kefeng Ren, Ph.D., Zhejiang University, [renkf@zju.edu.cn](mailto:renkf@zju.edu.cn)

Kefeng Ren is expert in biomaterial interface

Jin Wang, Ph.D., Southwest Jiaotong University, [wangjin@home.swjtu.edu.cn](mailto:wangjin@home.swjtu.edu.cn)

Jin Wang is expert in heart valve biomaterials

Nan Huang, Ph.D., Southwest Jiaotong University, [nhuang@263.net](mailto:nhuang@263.net)

Nan Huang is expert in heart valve biomaterials

Anqiang Sun, Ph.D., Beihang University, [saq@buaa.edu.cn](mailto:saq@buaa.edu.cn)

Anqiang Sun is expert in biomechanics

With best regards!

Sincerely,

Yunbing Wang, Ph.D.

Professor, Director of National Engineering Research Center for Biomaterials, Sichuan University, Chengdu, PR China 610064

E-mail: yunbing.wang@scu.edu.cn

Tel: 86-28-85415280

Fax: 86-28-85410246

Yang Lei, Ph.D.

Associate Professor, National Engineering Research Center for Biomaterials, Sichuan University, Chengdu, PR China 610064

Email: leiyang@scu.edu.cn; [leiyang2016@qq.com](mailto:leiyang2016@qq.com)

Tel: 86-15828387026
