# Supplementary material for: The study of dry biological valve crosslinked with a combination of carbodiimide and polyphenol
Source: Regen Biomater. 2020 Dec 3;8(1):rbaa049. doi: 10.1093/rb/rbaa049 (PMC7947589; doi:10.1093/rb/rbaa049)
Supplement: rbaa049_Supplementary_Data [file rbaa049_supplementary_data.zip › suppl_data/Response 200911-1608.docx]

**Response to reviewers’ comments**

Manuscript ID: RB-2020-085

Manuscript title: The study of dry biological valve crosslinked with a combination of carbodiimide and polyphenol

We thank the reviewers for their insightful comments and happy to see their enthusiasm for our studies. We have addressed their concerns in the revised manuscript and responded to the comments below in a point-by-point manner. The revised text is highlighted in yellow in the revised manuscript.

**Comments from Reviewer: 1**

**[Comment]**

In this study, a non-glutaraldehyde crosslinked dry biological valve material was prepared and tested, for a better performance of artificial valve. For the mechanical test part, have authors verfied if the material is isotropic or nonisotropic? If the material is nonisotropic, the stretch direction in the test should be stated.

**[Response]**

Thanks for the comments from the reviewer. The pericardial material is nonisotropic and the specific description is added in the experimental section. “The mechanical properties of the pericardium were non-isotropic, so the material used for uniaxial mechanical testing was in the direction of collagen fibers. The direction of the sample was confirmed under the light and magnifying glass. The supplement information had the methods and results of biaxial mechanical test, which further confirmed the nonisotropy of the material.”

**[Revision]**

**Materials and Methods**

The mechanical properties of the pericardium were non-isotropic, so the material used for uniaxial mechanical testing was in the direction of collagen fibers. The direction of the sample was confirmed under the light and magnifying glass. The supplement information had the methods and results of biaxial mechanical test, which further confirmed the nonisotropy of the material.

**Supplement Information**

Materials and Methods

*Biaxial tensile test*

Samples from EDC/CC treatment group was cut into 1 cm × 1 cm squares (N = 4). The fiber direction was made parallel or perpendicular to the edge. The sample was then placed on a rubber pad and secured to the tensile tester (BioTester, 10 N load cell; CellScale, Waterloo, Ontario, Canada) in PBS solution [1]. The tissue was passed through by the hooks of each side of the sample, and the four edges were evenly distributed. The horizontal was defined as the X direction and the vertical was defined as the Y direction. The force loading test protocol was used to maximize the load force (approximately 1500 N ~ 2500 N, depending on the material) while ensuring that the sample was not damaged. The force value loaded in X direction and Y direction remained 1:1. After the strain was derived by image tracking, the stress-strain curve of the sample was calculated.

Results

The biaxial stretching results show that the pericardium has different elastic modulus in X and Y perpendicular directions, indicating its non-isotropy (Figure S1).

**[Comment]**

Besides, the pre-crimping test would cause damage to the material in some certain directions. Have the authors considered the mechanical performance in different directions after pre-crimping? These two issues are suggested to be considered or discussed.

**[Response]**

In the pre-crimping test, the pericardial tissue was folded and squished together before being plugged into a catheter, so the material was squeezed and deformed in all directions to simulate the loading of the transcatheter biological valve. From the experimental results, the glutaraldehyde control group showed certain creases and damage in all directions after folding and pressing, without obvious anisotropy.

**[Revision]**

In the pre-crimping test, the pericardial tissue was folded and squished together before being plugged into a catheter, so the material was squeezed and deformed in all directions to simulate the loading of the transcatheter biological valve. From the experimental results, the glutaraldehyde control group showed certain creases and damage in all directions after folding and pressing, without obvious anisotropy.

**Comments from Reviewer: 2**

**[Comment]**

In this manuscript (RB-2020-085), the authors studied the crosslinking of pericardium through non-glutaraldehyde method. The EDC and polyphenol were used to crosslink pericardium. The authors showed that such crosslinked pericardium have good mechanical properties, anti-calcification, cytotoxicity, hemocompatibility. Therefore, the EDC/polyphenol crosslinking method could be used as a very good alternative for pericardium, which would be important for the development of the pericardium medical device. The overall study is clear and feasible, and the experimental data can support the author's conclusion. I would like to recommend it for publication. Still, English of the manuscript should be improved.

**[Response]**

Thanks for the comments from the reviewer. The English of the manuscript has been revised. All the authors have revised and confirmed the English spelling, grammar and other language problems.

**[Revision]**

**Abstract**

However, problems such as glutaraldehyde residue and calcification still exist in current commercial products. Non-glutaraldehyde crosslinked dry valve is an important strategy to solve those problems

The results showed that the comprehensive properties of EDC and curcumin crosslinked pericardium was superior to glutaraldehyde crosslinked pericardium, including unfolding property, anti-calcification, cytotoxicity, anticoagulant properties, mechanical properties, enzyme degradation resistance, and thermal shrinkage temperature. EDC and curcumin crosslinked dry pericardium could flatten after being folded at 40℃ for 3 days while glutaraldehyde crosslinked pericardium could not.

**Introduction**

Currently, commercial bioprosthetic heart valves are mostly made of glutaraldehyde crosslinked pericardia.

However, they still have a short lifespan of 10-15 years.

At the same time, the wet valve needs to be instantly cleaned and installed before the valve replacement operation.

Thus, steps such as preoperative cleaning and installation can be avoided, which can reduce the residual glutaraldehyde and shorten the preoperative preparation time of the instrument

Glutaraldehyde crosslinked dry valve could not flatten in water.

N-hydroxysuccinimide (NHS) could further enhance the crosslinking efficiency of EDC [2, 5]. EDC crosslinking can effectively enhance the stability of collagen, but the crosslinking effect on elastin is limited [2]. Polyphenolic compounds, including curcumin and procyanidins, can achieve pericardium crosslinking through hydrogen bonding [3-6].

**Results**

The pre-crimping test results showed that the dry valve in the glutaraldehyde treatment group could not completely flatten in water after being folded at 40℃ for 3 days, and the creasing was relatively obvious (Figure 1, supplemental video 1).

The results of the platelet adhesion test showed that there was more adhered platelet in the glutaraldehyde treatment group, and almost no adhered platelet was seen in the CC, EDC/CC, PC, or EDC/PCC treatment group (Figure 4).

**Discussion**

In addition, preassembly of biological valves’ damage still exists.

The formation of stable crosslinks by glutaraldehyde is formed by either the combination of two intermediate Schiff bases or the addition of another ε-amino group to the double bond [22]. Carbodiimide can crosslink neighboring carboxyl and amine groups and form amide bonds [23]. Carbodiimide crosslinked pericardia were softer. Our results confirmed that carbodiimide and curcumin group had lower modulus compared to the glutaraldehyde group (Figure 7B).

The maximum tensile strength is related to the quality, thickness, decellularization process and the crosslinking degree of the pericardial materials.

**[Comment]**

The Figure legends are too simple to understand the figure contents.

**[Response]**

All the legends have been expanded in detail to facilitate the reader's understanding.

**[Comment]**

The scale bars are missing in Figure 1 and 6.

**[Response]**

The scale bars were added in Figure 1 and 6 in revised manuscript.
